# The effect of percutaneous coronary intervention on habitual physical activity in older patients

**DOI:** 10.1186/s12872-016-0428-7

**Published:** 2016-12-03

**Authors:** Sarah J. Charman, Vincent T. van Hees, Louise Quinn, Joseph R. Dunford, Bilal Bawamia, Murugapathy Veerasamy, Michael I. Trenell, Djordje G. Jakovljevic, Vijay Kunadian

**Affiliations:** 1 Institute of Cellular Medicine, Faculty of Medical Sciences, Newcastle University, Newcastle upon Tyne, NE2 4HH UK; 2 Netherlands eScience Center, Amsterdam, The Netherlands; 3 Cardiothoracic Centre, Freeman Hospital, Newcastle upon Tyne Hospitals NHS Foundation Trust, Newcastle upon Tyne, UK

## Abstract

**Background:**

Given the ongoing burden of cardiovascular disease and an ageing population, physical activity in patients with coronary artery disease needs to be emphasized. This study assessed whether sedentary behaviour and physical activity levels differed among older patients (≥75 years) following percutaneous coronary intervention (PCI) for acute coronary syndrome (ACS) consisting of ST-segment elevation myocardial infarction (STEMI) and non STEMI (NSTEMI) versus an elective admission control group of stable angina patients.

**Methods:**

Sedentary behaviour and physical activity were assessed over a 7-day period using wrist-worn triaxial accelerometers (GENEActiv, Activinsights Ltd, UK) in 58 patients following PCI for, STEMI (*n* = 20) NSTEMI (*n* = 18) and stable angina (*n* = 20) upon discharge from a tertiary centre. Mean ± Standard deviation age was 79 ± 4 years (31% female).

**Results:**

STEMI and NSTEMI patients spent more time in the low acceleration category (0–40 mg) reflecting sedentary time versus stable angina patients (1298 ± 59 and 1305 ± 66 vs. 1240 ± 92 min/day, *p* < 0.05). STEMI and NSTEMI patients spent less time in the 40–80 mg acceleration category reflecting low physical activity versus stable angina patients (95 ± 35 and 94 ± 41 vs. 132 ± 50 min/day, *p* < 0.05). Stable angina patients spent more time in the higher acceleration categories (80–120 and 120–160 mg) and moderate-to-vigorous physical activity (defined as 1 and 5 min/day bouts) versus NSTEMI patients (*p* < 0.05). For acceleration categories ≥160 mg, no differences were observed.

**Conclusions:**

Patients presenting with ACS and undergoing PCI spent more time in sedentary behaviour compared with stable angina patients.

## Background

Cardiovascular disease is a leading cause of death in both the United Kingdom (UK) and the United States of America (USA) [1, 2]. In the UK, cardiovascular disease accounted for 180,000 deaths in 2010, 80,000 of which were caused by coronary artery disease (CAD) [1]. In the USA, cardiovascular disease resulted in 1 in 3 deaths in 2009 [2]. Sedentary behaviours, such as the amount of time spent sitting are associated with increased risk of cardiovascular disease and all-cause mortality, independent of physical activity [3]. Regular participation in physical activity is associated with a decreased risk of CAD [1] and a dose-dependent response relationship has been observed in patients living with CAD, leading to reduced risk of all-cause mortality [4]. Current trends in most developed countries highlight a shift in ageing coupled with individual’s performing less physical activity [5, 6]. In England, only 9% of men and 6% of women aged 75+ years achieved the recommended physical activity guidelines [7], whereas in the USA, 9.9% of men and 4.8% of women aged 75+ years met both the aerobic and muscle strengthening guidelines [8]. These statistics highlight that the majority of our ageing population are not engaging in the necessary levels of physical activity to benefit health in the setting of cardiovascular disease [1, 7, 8].

Given that our ageing population spend a substantial amount of time sedentary [9], one study has demonstrated that elderly patients (>65 years) reporting a sedentary lifestyle after admittance to hospital with CAD or congestive heart failure had nearly four times greater risk of mortality compared to non-sedentary elderly patients [10]. Increasing physical activity level can improve cardiovascular risk factors, quality of life and exercise capacity in older patients with CAD [11]. It has also been suggested that intensity of physical activity is associated with reduced cardiovascular morbidity and mortality in older patients [12].

It has recently been suggested that older patients with CAD are underrepresented in clinical research [13]. Patients presenting for treatment procedures such as percutaneous coronary intervention (PCI) following acute coronary syndrome is increasing in older patients [14]. The findings by Presutti et al. [15] highlight that this procedure in the elderly may be beneficial and should not be denied in this frail patient cohort. Whether increased levels of physical activity can prevent cardiovascular events in older patients with CAD is poorly understood. Furthermore, sedentary behaviour and physical activity have not previously been defined, limiting our abilities in designing interventions to improve clinical outcomes in older patients with CAD. Therefore, the present study aimed to evaluate the physical activity patterns following PCI in older patients with CAD. In particular we attempted to objectively determine differences in time spent in categories of wrist acceleration reflecting both sedentary behaviour and physical activity in older patients following PCI.

## Methods

### Sample

A total of 65 patients aged 75 years and older were recruited. All patients had their sedentary behaviour and physical activity monitored upon discharge from the tertiary centre. In addition, the research team collected patient clinical baseline characteristics, baseline blood tests and co-morbidities.

### Measurements

Patient’s age (years) was recorded as a decimal value. Standing height (to the nearest 0.1 cm) and body weight (to the nearest 0.1 kg) were measured using the SECA stadiometer and scales (North East Weighing & Calibration Ltd.). Body mass index (BMI) was calculated using the equation: BMI = body mass (kg) ÷ stature^2^ (m^2^). Patient clinical characteristics included PCI (single or multi vessel), access for PCI (radial or femoral), prior myocardial infarction (yes or no), prior PCI (yes or no), prior coronary artery bypass graft (yes or no), hypertension (yes or no), current smoker (yes or no), family history of ischemic heart disease (yes or no), Type 2 diabetes (yes or no), prior peripheral vascular disease (yes or no), prior history of TIA/stroke (yes or no), history of congestive heart failure (yes or no), history of liver disease (yes or no), history of malignancy (yes or no), history of dementia (yes or no), creatinine (mg/dL), haemoglobin (g/dL) and peak troponin value during hospitalisation (ng/dL).

Wrist-worn accelerometers (GENEActiv, Activinsights Ltd, United Kingdom) were used to monitor sedentary behaviour and physical activity in patients following PCI for stable angina, STEMI and NSTEMI following discharge from the tertiary centre. The accelerometer was pre-programmed to start recording on disconnect from the computer and was subsequently attached to the patients wrist (patient preference and worn on either right or left wrist) by a member of the research team. The accelerometer was worn continuously for a period of 7 days in free-living conditions. Patients were required to have worn the monitor for at least five of the seven monitoring days. From the 65 patients meeting the inclusion criteria for the study, a total of 58 patient’s data were included in the final analysis with three patients excluded due monitor removal (*n* = 3, STEMI), two patients dropped out as they did not like wearing the monitor (*n* = 1, STEMI; *n* = 1, NSTEMI) and two patients deceased prior to discharge from the tertiary centre (*n* = 1, STEMI; *n* = 1, NSTEMI). After completion of monitoring, patients were called at their home address to remind them to return the accelerometer to the tertiary centre by post.

Accelerometer data was processed in R (www.cran.r-project.org) using R-package GGIR [16, 17]. Signals were inspected and corrected for calibration error [18]. The first and last hour of the measurement were excluded as they are expected to be influenced by the monitor distribution and collection procedure. Only days with at least 16 h of valid data were retained for further analysis. Next, the average magnitude of wrist acceleration per 5 s epoch was calculated with metric ENMO as previously described [17]. The output from metric ENMO is in m*g* (1 m*g* = 0.001 *g* = 0.001 × 9.8 m/s^2^ = 0.001 × gravitational acceleration) [17]. Monitor non-wear was detected as described previously [17] and replaced by the average accelerometer data on similar time points on different days of the measurement [19, 20]. The imputation procedure as used is effectively the same as taking the average of all available data weighted by the number of available data points per time of the day. In contrast, taking the plain average of all available data points would cause unequal weighting of periods within the 24 cycle and result in an unstandardized estimate of physical activity. The resulting time series were used to derive time spent in acceleration categories per day. For this study we calculated time spent in the following acceleration categories: Category (0–40 mg) reflects inactive behaviour; categories (40–80 mg) and (80-120 mg) reflect a mixed zone between active and inactive behaviour (e.g. sitting while moving arms, or slow walking while keeping hands still); categories (120–160 mg) and higher reflect moderate and vigorous physical activity [21]. Moderate-to-vigorous physical activity was calculated using a ≥100 mg cut-off with 1 and 5 min/day bouts determined [21]. Bouts of moderate-to-vigorous physical activity are identified as all 1 or 5 min time windows that start with a 5 s epoch value equal or higher than 100 mg and for which 80% of subsequent 5 s epoch values are equal to or higher than the 100 mg threshold.

### Statistical analysis

Prior to analysis, procedures for checking violations of test assumptions were conducted. All acceleration categories (0–40, 40–80, 80–120, 120–160, 160–200, 200–240, 240–280, 280–320 mg, respectively) and moderate-to-vigorous physical activity (1 and 5 min/day bouts) were non-normally distributed and were subsequently square-root transformed for the main analyses. Demographic and clinical baseline characteristics were presented as a percentage or mean ± SD. One-way analysis of variance (ANOVA) assessed between group differences in demographic baseline characteristics for the three CAD hospitalisation groups (stable angina, STEMI and NSTEMI) with follow up Bonferroni tests conducted. Chi-square tests were performed on the categorical data for the clinical baseline characteristics. A Mann-Whitney U test and Kruskal-Wallis H tests were performed on the continuous data for the clinical baseline characteristics. One-way ANOVA assessed between group differences in acceleration categories and moderate-to-vigorous physical activity (1 and 5 min/day bouts) for the three CAD hospitalisation groups (stable angina, STEMI and NSTEMI) with follow up Bonferroni tests conducted. All data were analysed using SPSS, version 21 (SPSS, Inc., Chicago, IL, USA). The level of significance was set at *p* < 0.05.

## Results

Demographic and clinical baseline characteristics are detailed in Tables 1 and 2, respectively. Mean ± standard deviation age for all patients was 79 ± 4 years (36% of sample ≥80 years) with 31% of patients being female. NSTEMI patients were significantly older than stable angina patients (*p* < 0.05). No other significant differences were found between the three CAD hospitalisation groups.

**Table 1:** Demographic characteristics (all patients and coronary heart disease hospitalisation groups)

Variable	Total	Stable Angina	STEMI	NSTEMI
	*N* = 58	*N* = 20	*N* = 20	*N* = 18
Age (mean ± SD)	79 ± 4	78 ± 3	78 ± 2	81 ± 6*
<80 years (%)	64	75	70	44
≥80 years (%)	36	25	30	56
Sex n (% of sample female)	18 (31)	6 (30)	5 (25)	7 (39)
Standing height (cm)	168 ± 9	167 ± 9	169 ± 9	168 ± 10
Body weight (kg)	75 ± 14	74 ± 15	77 ± 15	74 ± 11
Body mass index (kg/m^2^)	27 ± 4	26 ± 4	27 ± 5	26 ± 4

Abbreviations: *STEMI* ST-segment elevation myocardial infarction, *NSTEMI* non-ST-segment elevation myocardial infarction. *p < 0.05

**Table 2:** Clinical characteristics (all patients and coronary artery disease hospitalisation groups)

Variable	Total	Stable Angina	STEMI	NSTEMI
	*N* = 58	*N* = 20	*N* = 20	*N* = 18
Single vessel PCI n (%)	40 (69)	11 (55)	20 (100)	9 (50)
Multi-vessel PCI n (%)	18 (31)	9 (45)	0 (0)	9 (50)
Radial arterial access for PCI n (%)	49 (85)	17 (85)	16 (80)	16 (89)
Femoral arterial access for PCI n (%)	9 (15)	3 (15)	4 (20)	2 (11)
Prior myocardial infarction n (%)	19 (33)	10 (50)	2 (10)	7 (39)
Prior PCI n (%)	14 (24)	9 (45)	1 (5)	4 (22)
Prior CABG n (%)	7 (12)	2 (10)	0 (0)	5 (28)
Hypertension n (%)	39 (67)	14 (70)	13 (65)	12 (67)
Current smoker n (%)	10 (17)	1 (5)	4 (20)	5 (28)
Family history of IHD n (%)	20 (35)	7 (35)	5 (25)	8 (44)
Type 2 diabetes n (%)	12 (21)	4 (20)	4 (20)	4 (22)
Prior PVD n (%)	4 (7)	1 (5)	0 (0)	3 (17)
Prior history of TIA/stroke n (%)	11 (19)	4 (20)	1 (5)	6 (33)
History of CHF n (%)	2 (3)	2 (10)	0 (0)	0 (0)
History of liver disease n (%)	0 (0)	0 (0)	0 (0)	0 (0)
History of malignancy n (%)	8 (14)	4 (20)	3 (15)	1 (6)
History of dementia n (%)	0 (0)	0 (0)	0 (0)	0 (0)
Creatinine (mg/dL)	102 ± 28	103 ± 29	94 ± 23	109 ± 30
Haemoglobin (g/dL)	13 ± 2	13 ± 2	13 ± 3	13 ± 2
Peak troponin value during hospitalisation (ng/dL)	1066 ± 1644	N/A	2418 ± 2037	680 ± 637
Time from presentation to PCI (hours)	-	N/A	1 ± 1	107 ± 97
Time from PCI to discharge from hospital (hours)	34 ± 20	18 ± 13	49 ± 12	35 ± 22

Abbreviations: *STEMI* ST-segment elevation myocardial infarction, *NSTEMI* non-ST-segment elevation myocardial infarction, *PCI* percutaneous coronary intervention, *CABG* coronary artery bypass graft, *IHD* ischemic heart disease, *PVD* peripheral vascular disease, *CHF* congestive heart failure

Patients presented with stable angina (*n* = 20), STEMI (*n* = 20) or NSTEMI (*n* = 18) when admitted to hospital for PCI, with the majority having single vessel PCI performed (69%). However, when separated into CAD hospitalisation groups, a significant association was found with 100% of STEMI patients having a single vessel procedure whilst 50% and 45% of NSTEMI and stable angina patients had a multi vessel procedure, respectively, *χ* ^2^ (2, *N* = 58) = 13.85, *p* < 0.01. The majority of patients had a radial arterial access procedure for PCI (*n* = 49, 85% of sample) with 9 patients (stable angina (*n* = 3), STEMI (*n* = 4) and NSTEMI (*n* = 2)) having a femoral procedure. Prior PCI was reported in 24% of the sample. When separated into the three CAD hospitalisation groups, results revealed that only 5% of STEMI had a prior PCI whereas 22% of NSTEMI and 45% of stable angina patients had a prior PCI, *χ* ^2^ (2, *N* = 58) = 8.79, *p* < 0.05. Prior myocardial infarction was reported in 33% of the sample with 50% of stable angina patients suffering a prior myocardial infarction compared with only 10% of STEMI patients and 39% NSTEMI patients, *χ* ^2^ (2, *N* = 58) = 7,71, *p* < 0.05. Prior coronary artery bypass graft was reported in 12% of the sample with 100% of STEMI patients not having a prior coronary artery bypass graft whereas 28% of NSTEMI and 10% of stable angina patients having this procedure previously, *χ* ^2^ (2, *N* = 58) = 7.01, *p* < 0.05.

No other associations were reported between CAD hospitalisation groups and the categorical variables. Prior peripheral vascular disease in 7% of the sample, hypertension was reported in 67% of the sample, 17% were current smokers and 21% had Type 2 diabetes. History of congestive heart failure (3%) and history of malignancy (14%) were reported in this sample but no patients reported a history of either liver disease or dementia. Mean peak troponin value during hospitalisation for all patients (STEMI and NSTEMI only) was 1066 ± 1644 ng/dL with STEMI patients having a significantly higher score than NSTEMI patients (*U* = 70, *p* < 0.01). Mean creatinine and mean haemoglobin for all patients were 102 ± 28 mg/dL and 13 ± 2 g/dL, respectively with no significant differences in reported values found between the three CAD hospitalisation groups for either measures.

The time from presentation to PCI is reported in Table 2 with a significant difference found between the two ACS hospitalisation groups (STEMI vs. NSTEMI), *χ* ^2^ (1) = 21.07, *p* < 0.001, with a mean rank time score of 11.65 for STEMI and 28.22 for NSTEMI. The time from PCI to discharge from hospital is reported in Table 2 with stable angina patients spending a mean time of 18 ± 13 h in hospital from procedure to discharge. There was a statistically significant difference in PCI to discharge from hospital between the three CAD hospitalisation groups, *χ* ^2^ (2) = 33.12, *p* < 0.001, with a mean rank time score of 44.68 for STEMI, 29.92 for NSTEMI and 13.95 for stable angina. Significant differences were observed between times for all three CAD hospitalisation groups (STEMI vs. NSTEMI, *χ* ^2^ (1) = 12.41, *p* < 0.001; NSTEMI vs. stable angina, *χ* ^2^ (1) = 14.01, *p* < 0.001; STEMI vs. stable angina, *χ* ^2^ (1) = 24.51, *p* < 0.001).

**Fig. 1:**
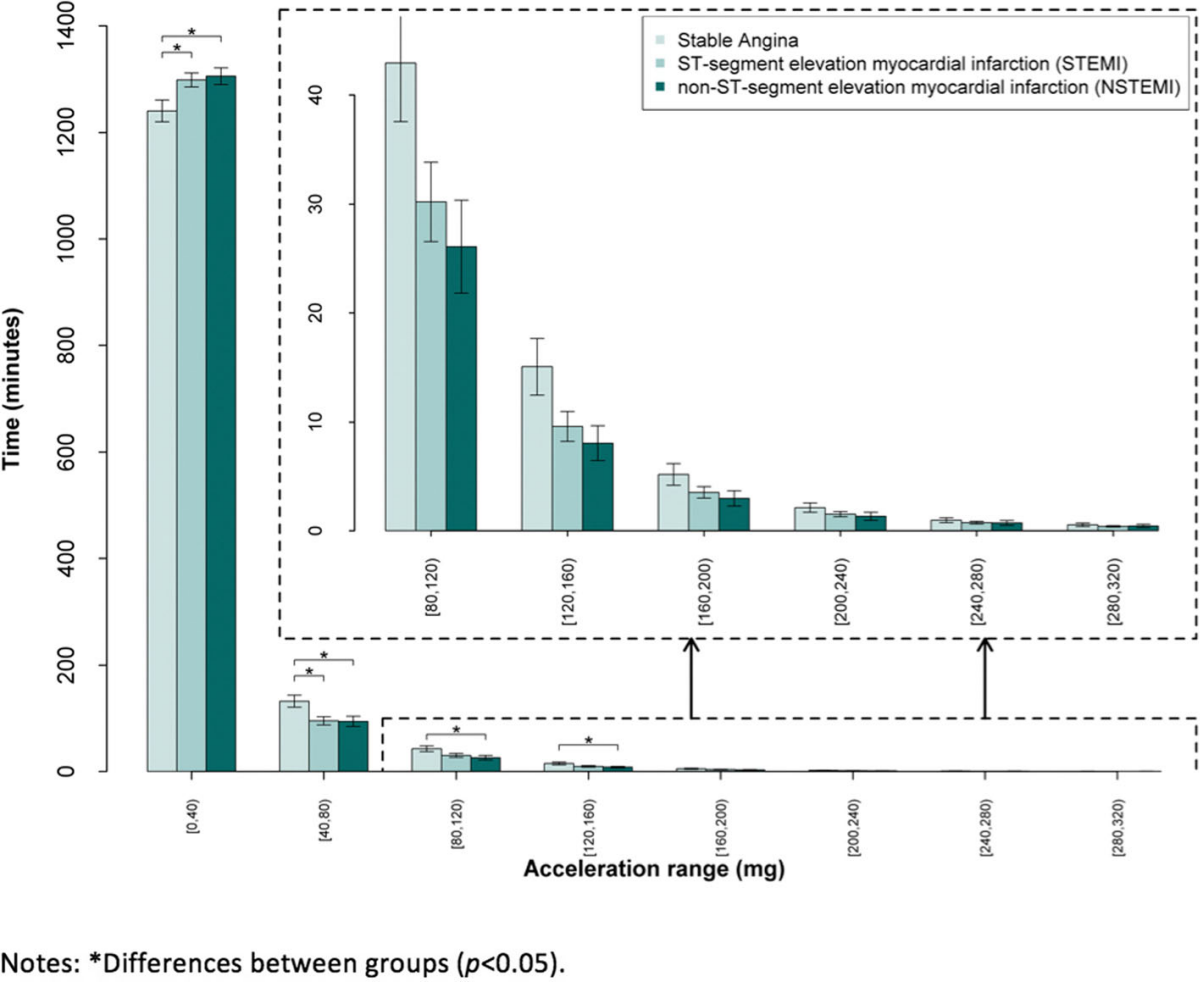
Time spent in acceleration categories for all coronary artery disease hospitalisation groups

Acceleration categories associated with sedentary behaviour and physical activity for all patients is presented in Table 3 and Fig. 1. STEMI and NSTEMI patients spent significantly more time in the lowest acceleration category (0–40 mg) than stable angina patients (*p* < 0.05). STEMI and NSTEMI patients spent significantly less time than stable angina patients in the 40–80 mg acceleration category (*p* < 0.05). These trends continued for acceleration categories 80–120 mg (*p* < 0.05) and 120–160 mg (*p* < 0.05) but only between NSTEMI and stable angina patients. For the higher acceleration categories (>160 mg), no differences between the groups were observed. Differences between-groups were observed for time spent in moderate-to-vigorous physical activity. Stable angina patients spent more time than NSTEMI patients in moderate-to-vigorous physical activity, both when defined as 1 min/day (*p* < 0.05) and 5 min/day bouts (*p* < 0.05).

**Table 3:** Time (minutes) spent in acceleration categories and moderate-to-vigorous physical activity bouts (all patients and coronary artery disease hospitalisation groups)

Acceleration categories	Total	Stable Angina	STEMI	NSTEMI
	*N* = 58	*N* = 20	*N* = 20	*N* = 18
0–40 mg (all days)^a^	1280 ± 78	1240 ± 92	1298 ± 59	1305 ± 66
40–80 mg (all days)^a^	108 ± 45	132 ± 50	95 ± 35	94 ± 41
80–120 mg (all days)^a^	33 ± 21	43 ± 24	30 ± 16	26 ± 18
120–160 mg (all days)^a^	11 ± 9	15 ± 12	10 ± 6	8 ± 7
160–180 mg (all days)^a^	4 ± 3	5 ± 4	4 ± 2	3 ± 3
200–240 mg (all days)^a^	2 ± 2	2 ± 2	2 ± 1	1 ± 2
240–280 mg (all days)^a^	1 ± 1	1 ± 1	1 ± 1	1 ± 1
280–320 mg (all days)^a^	0 ± 1	1 ± 1	0 ± 0	0 ± 1
MVPA (1 min/day bouts)^a^	10 ± 13	15 ± 18	7 ± 6	6 ± 9
MVPA (5 min/day bouts)^a^	3 ± 6	6 ± 10	3 ± 4	1 ± 3
Average wrist acceleration (ENMO, mg)	15 ± 5	18 ± 6	14 ± 4	14 ± 4
Time accelerometer worn (days)	7 ± 0	7 ± 1	7 ± 0	7 ± 0

Abbreviations: *STEMI* ST-segment elevation myocardial infarction, *NSTEMI* non-ST-segment elevation myocardial infarction, *MVPA* moderate-to-vigorous physical activity. ^a^Untransformed values shown

## Discussion

This is the first study to define differences in time spent in acceleration categories reflecting sedentary behaviour and physical activity using wrist-worn accelerometers in older patients (≥75 years) following PCI presenting with ACS consisting of STEMI and NSTEMI or elective admissions presenting with stable angina. Major findings suggest that patients presenting with ACS and undergoing PCI spent more time in acceleration categories reflecting sedentary behaviour compared to stable angina patients (0–40 mg category) upon discharge from hospital. STEMI and NSTEMI patients spent less time in acceleration categories reflecting low physical activity (40–80 mg) when compared to stable angina patients. Differences were observed between groups for stable angina and NSTEMI patients for acceleration categories reflecting low to moderate physical activity (80–120 mg and 120–160 mg) and for moderate-to-vigorous physical activity (1 and 5 min/day bouts) with the former spending more time in the higher acceleration categories.

Our data demonstrate that our elderly population spent over 20 h/day either sedentary or sleeping based on the lowest acceleration category (STEMI = 1298 ± 59; NSTEMI = 1305 ± 66; stable angina = 1240 ± 92 min/day, respectively). The mean difference in time spent in the lowest acceleration category equated to approximately an hour more time spent sedentary or sleeping in ACS patients (STEMI and NSTEMI) versus stable angina patients. Differences found between time spent in the lowest acceleration category (0–40 mg) between stable angina patients, STEMI and NSTEMI patients aged ≥75 years has not been previously compared in patients with CAD. However, positive associations have been found between sedentary behaviour and cardiometabolic risk factors after adjustment for moderate-to-vigorous physical activity in older adults (≥65 years) [22]. Previous studies have highlighted increased mortality risk related to greater amounts of time spent sedentary, but have generally employed subjective methods to evaluate activity levels, such as questionnaires [10, 23].

In one such study, older patients (≥65 years of age) categorised as sedentary had nearly a 4-fold increased mortality rate to non-sedentary patients following hospitalisation for CAD or congestive heart failure [10]. Similarly, in a younger population of men (≥45 years of age), patients in the self-reported low physical activity category had a significantly greater incidence of heart failure than those reporting medium and high physical activity categories during a 7.8 year follow-up [23]. Sedentary behaviours such as riding in a car for >10 h/week were also associated with a 48% increased risk of cardiovascular disease mortality in men during a 21 year follow-up [24]. All of these studies demonstrate the adverse health consequences of sedentary behaviour longitudinally. Whether sedentary behaviour is associated with increased cardiovascular disease morbidity and mortality in older CAD patients will be subject to a separate study, however the present data suggests for the first time that the majority of older patients following PCI spend long periods engaging in sedentary behaviour and sleep.

Distinct differences were also observed in time spent in acceleration categories reflecting low to moderate physical activity (80–120 mg and 120–160 mg), with stable angina patients spending 17 and 7 more minutes/day in the 80–120 mg and 120–160 mg categories, respectively versus NSTEMI patients. Stable angina patients spent significantly more time than NSTEMI patients in moderate-to-vigorous physical activity as defined in the 1 (15 ± 18 vs. 6 ± 9 min/day bouts, *p* < 0.05) and 5 (6 ± 10 vs. 1 ± 3 min/day bouts, *p* < 0.05) minutes/day bouts. Previously, comparison across CAD hospitalisation groups has observed coronary artery bypass graft patients to be more active than PCI patients (*P* = 0.033) over follow-up (2, 6 and 12 months) [25]. However, in this study differences were observed between stable angina and NSTEMI groups, both of which had undergone PCI.

Overall, patients spent little time in moderate-to-vigorous physical activity based on 1 or 5 min/day bouts (all patients = 10 ± 13 and 3 ± 6 min/day respectively). A small number of patients accumulated ≥30 min/day of moderate-to-vigorous physical activity in the 1 (*n* = 4) and 5 (*n* = 1) minutes/day bouts. Consequently, the majority of patients did not meet the recommended physical activity guidelines of 150 min/week of at least moderate intensity physical activity for older adults [26]. It is recommended that patients attend cardiac rehabilitation upon discharge following hospitalisation for CAD [1], which can lead to decreased mortality risk even in older patients (≥65 years) with 1 and 5 year follow-up in mortality reductions of 58 and 34%, respectively, compared to non-cardiac rehabilitation users [27]. Indeed, one weekly exercise session (versus no activity) is associated with lowering all-cause mortality in both men and women with established coronary heart disease [28]. However, the type of exercise programme may have a significant effect on health outcomes, with Munk et al. [29] demonstrating that high-intensity interval training (versus moderate continuous training) produced a significant reduction in late luminal loss in the stented coronary segment in patients (59.2 ± 9.5 years of age) following PCI for stable and unstable angina. However, little information is available on the effect of increased physical activity on cardiovascular disease morbidity and mortality on older patients (>75 years of age) with CAD.

Our data suggests that there were no significant differences between STEMI and NSTEMI patients for either the time spent in the lowest acceleration category (0–40 mg) or categories reflecting low to moderate physical activity (40–80 mg, 80–120 mg, 120–160 mg). Likewise, no differences were observed between these two groups for moderate-to-vigorous physical activity based on the 1 and 5 min/day bouts.

This present study has a number of strengths, which include the comparison of objectively measured sedentary behaviour and physical activity across three elderly patient groups directly following discharge from hospital for PCI treatment. However, this study is not without limitations, and these include the cross-sectional nature of the study, which mean causal inferences cannot be made. Given the age of the patients, it was difficult to recruit a control group free of disease, therefore, elective admissions for PCI presenting with stable angina was deemed a control group for comparison with ACS groups. The small sample size also makes it difficult to generalise these findings at a population level. In addition, it has been previously reported that radial arterial access for PCI can reduce the risk of mortality [30] and present fewer problems with mobility and self-care [31] versus a femoral procedure in STEMI patients. However, no significant differences in the acceleration categories or moderate-to-vigorous physical activity (1 and 5 min/day bouts) were found between patients following radial versus femoral arterial access for PCI. In this study the majority of patients had a radial arterial access for PCI (*n* = 49), which meant comparisons between access groups for the sedentary behaviour and physical activity variables were limited.

## Conclusions

The findings from this study highlight that the acceleration category reflecting sedentary behaviour was generally higher in ACS (STEMI and NSTEMI) patients versus stable angina patients whereas time spent in acceleration categories reflecting low to moderate physical activity and moderate-to-vigorous physical activity bouts were greater among stable angina patients compared to the NSTEMI patients. Observations of this kind could be used to develop future intervention based studies to demonstrate the starting level of sedentary time and physical activity in elderly (≥75 years) patients post PCI in three specific CAD hospitalisation groups. Longitudinal observational data is needed to determine associations between changes in sedentary behaviour and physical activity and cardiovascular disease morbidity and mortality in older patients with CAD.

## Abbreviations

ACS: Acute coronary syndrome
ANOVA: Analysis of variance
BMI: Body mass index
CABG: Coronary artery bypass graft
CAD: Coronary artery disease
CHF: Congestive heart failure
IHD: Ischemic heart disease
MVPA: Moderate-to-vigorous physical activity
NSTEMI: Non-ST-segment elevation myocardial infarction
PCI: Percutaneous coronary intervention
PVD: Peripheral vascular disease
STEMI: ST-segment elevation myocardial infarction
UK: United Kingdom
USA: United States of America
